# Increased risk of herpes zoster in patients with psoriasis: A population-based retrospective cohort study

**DOI:** 10.1371/journal.pone.0179447

**Published:** 2017-08-22

**Authors:** Shin-Yi Tsai, Hsuan-Ju Chen, Chon-Fu Lio, Hui-Ping Ho, Chien-Feng Kuo, Xiaofeng Jia, Chi Chen, Yu-Tien Chen, Yi-Ting Chou, Tse-Yen Yang, Fang-Ju Sun, Leiyu Shi

**Affiliations:** 1 Department of Laboratory Medicine, Mackay Memorial Hospital, Taipei City, Taiwan; 2 Department of Medicine, Mackay Medical College, New Taipei City, Taiwan; 3 Department of Health Policy and Management, Johns Hopkins University Bloomberg School of Public Health, Baltimore, Maryland, United States; 4 Management Office for Health Data, China Medical University Hospital, Taichung City, Taiwan; 5 College of Medicine, China Medical University, Taichung City, Taiwan; 6 Centro Hospitalar Conde de São Januário, Macao; 7 Department of Infectious Disease, Mackay Memorial Hospital, Taipei City, Taiwan; 8 Department of Neurosurgery, University of Maryland School of Medicine, Baltimore, Maryland, United States; 9 Department of Biomedical Engineering, Johns Hopkins University, Baltimore, Maryland, United States; 10 Department of Psychiatry, University of Oxford, Oxford, United Kingdom; 11 Molecular and Genomic Epidemiology Center, China Medical University Hospital, Taichung City, Taiwan; 12 Division of Nephrology, Department of Internal Medicine, Changhua Christian Hospital, Changhua County, Lugang Town, Taiwan; 13 Department of Medical Research, Mackay Memorial Hospital, Taipei City, Taiwan; 14 Mackay Junior College of Medicine, Nursing and Management, Taipei City, Taiwan; Katholieke Universiteit Leuven Rega Institute for Medical Research, BELGIUM

## Abstract

**Objectives:**

The risk of herpes zoster (HZ) between patients with psoriasis receiving and not receiving systemic therapy has received increasing attention. This study investigated the association of psoriasis with the risk of HZ.

**Methods:**

We conducted a population-based retrospective cohort study by using the Taiwan National Health Insurance Research Database. The psoriasis cohort consisted of 4077 patients with newly diagnosed psoriasis between 2000 and 2006. Each patient with psoriasis was frequency-matched with four people without psoriasis, by sex, age and index year. (nonpsoriasis cohort; 16308 subjects). Patients who received systemic therapy were classified as having severe psoriasis, whereas those who did not receive systemic therapy were classified as having mild psoriasis. The Cox proportional hazards regression analysis was conducted to estimate the association between psoriasis and HZ risk.

**Results:**

The overall incidence density rate of HZ in the psoriasis cohort than in the nonpsoriasis cohort (4.50 vs. 3.44 per 1,000 person-years), with a multivariable Cox proportional hazards model measured adjusted HR of 1.29 [95% confidence interval (CI) = 1.07–1.56]. In additional, compared with the nonpsoriasis cohort, the risk of HZ was higher in the severe psoriasis cohort than in the nonpsoriasis cohort (adjusted hazard ratio [HR], 1.61; 95% confidence interval [CI], 1.15–2.27). The comparison between psoriasis and nonpsoriasis cohorts revealed a greatest magnitude risk of HZ in women (adjusted HR, 1.36; 95% CI, 1.04–1.79), study participants in the age group of 20–39 years (adjusted HR, 1.77; 95% CI, 1.17–2.66), and study participants without any comorbidities (adjusted HR, 1.37; 95% CI, 1.02–1.84).

**Conclusions:**

Our results suggest that psoriasis is associated with an increased risk of HZ, which involves differences in sex and age. Although systemic therapy may have a major role in the risk of HZ, the intrinsic factors of psoriasis cannot be excluded.

## Introduction

Psoriasis is clinically diagnosed by appearance and is characterized by erythematous scaly plaques along with pain and pruritus.[1] The prevalence of psoriasis ranges from 0% to 2.1% in children and from 0.91% to 8.5% in adults worldwide.[2] Histologically, it is characterized as a high turnover rate of epidermal cells leading to the hyperproliferation of keratinocytes in the epidermis. Genetic and environmental factors resulting in immunological disturbance appear to have crucial roles in psoriasis development.[3] In addition to skin lesions, psoriatic arthritis, which most often affects finger and toe joints, is a common manifestation of immune-mediated inflammation involving the surrounding connective tissue. Guideline therapies include topical treatment and systemic therapy with methotrexate, cyclosporine, phototherapy (narrowband and broadband ultraviolet B [UVB] and psoralen and ultraviolet A [PUVA]), oral retinoids, and biological agents.[4]

Viral infections commonly occur in patients with compromised immune systems. The dissemination of varicella-zoster virus (VZV) has been investigated in several patient groups, such as in those with dermatomyositis and polymyositis, inflammatory bowel disease, systemic lupus erythematosus, and rheumatoid arthritis. These studies have reported an association of immune dysfunction with an increased risk of herpes zoster (HZ).[5–8] Although numerous molecular pathways have clarified the mechanism underlying this association, immunosuppressants may have a crucial role in this association. Some studies have reported a relationship between systemic antipsoriatic treatments and VZV dissemination.[9, 10] However, the results of these studies vary. The present study investigated the association of psoriasis with HZ under different severities defined according to treatment selection (no systemic therapy for the mild group and systemic therapy intervention for the severe group). We conducted a population-based retrospective cohort study by using the Taiwan National Health Insurance Research Database (NHIRD).

## Methods

### Data source

In March 1995, the Taiwan government implemented the National Health Insurance (NHI) program. This program has covered approximately 99% of 23.75 million individuals in Taiwan since 1999. By the end of 2014, the NHI program covered more than 99.9% of the Taiwanese population. In this study, we used the Longitudinal Health Insurance Database 2000 (LHID2000), which contains the original claims data of 1 000 000 individuals randomly sampled from the 2000 Registry for Beneficiaries of the NHIRD. The LHID2000 is a representative dataset of the entire population. The datasets of this study consisted of registry for beneficiaries and ambulatory and inpatient care claims data from the NHIRD between 1996 and 2011, which was released by the Taiwan National Health Research Institutes. All claims data related to healthcare services, including beneficiaries’ demographics, clinical visit dates, prescription details, and disease diagnoses were coded according to the International Classification of Diseases, Ninth Revision, Clinical Modification (ICD-9-CM) criteria, and have been collected and encrypted in the NHIRD. This study was approved by the Research Ethics Committee of China Medical University (CMUH104-REC2-115) and the Institutional Review Board of MacKay Memories Hospital (16MMHIS074).

### Study population

Patients who were aged more than 20 years, were diagnosed with psoriasis (ICD-9-CM 696) between January 1, 2000, and December 31, 2006, and had no history of HZ (ICD-9-CM 053) before enrollment were included in the psoriasis cohort. The date of psoriasis diagnosis was used as the index date. For each patient with psoriasis, 4 patients without psoriasis and HZ diagnoses before enrollment were randomly selected from the LHID2000; frequency-matched by sex, 5-year age interval, and index year; and included in the nonpsoriasis cohort.

Patients who were prescribed aggressive therapy after psoriasis diagnosis (namely phototherapy, including UVB and PUVA, and immunomodulatory therapy, including methotrexate, azathioprine, cyclosporin, oral retinoids, hydroxyurea, mycophenolate mofetil, tacrolimus, etanercept, adalimumab, and ustekinumab) were classified as having severe psoriasis. Patients who were not prescribed systemic or biological treatment or phototherapy for psoriasis were classified as having mild psoriasis.[11]

### Covariates and outcomes

The medical records of comorbidities such as diabetes mellitus (DM, ICD-9-CM 250); hyperlipidemia (ICD-9-CM 272); hypertension (ICD-9-CM 401–405); heart failure (HF, ICD-9-CM 428); vasculitis (ICD-9-CM 446 and 447.6); diffuse diseases of connective tissue(ICD-9-CM 710), including systemic lupus erythematosus(ICD-9-CM 710.0)[12], systemic sclerosis(ICD-9-CM 710.1)[13], sicca syndrome [13](ICD-9-CM 710.2), dermatomyositis (ICD-9-CM 710.3)[7], polymyositis (ICD-9-CM 710.4) [7], specified diffuse diseases of connective tissue (ICD-9-CM 710.8), unspecified diffuse connective tissue disease (ICD-9-CM 710.9)[13]; renal disease (ICD-9-CM 580–589); cancer (ICD-9-CM 140–208); inflammatory bowel disease (IBD; ICD-9-CM 555 and 556); chronic obstructive pulmonary disease (COPD, ICD-9-CM 491, 492, and 496), and asthma (ICD-9-CM 493) were obtained before the index date. We defined prescriptions of steroid use (including oral steroid and IV steroid) as those being prescribed for continuous 30 days during the period of follow-up.

The primary outcome was the occurrence of HZ (ICD-9-CM 053), which was determined through record linkage with the outpatient and inpatient claims data of the NHIRD. All patients were followed from the index date until the diagnosis of HZ, withdrawal from the NHI program, or the end of 2011, whichever occurred first.

### Statistical analysis

Summary statistics are expressed as frequencies and percentages for categorical variables and as means and standard deviations (SD) for continuous variables, as appropriate. Pearson’s chi-square test and Student *t* test were used to compare categorical and continuous variables between the psoriasis and nonpsoriasis cohorts, respectively. The incidence density rate of HZ was calculated by dividing the number of newly diagnosed HZ cases by person-years at risk for each group according to sex, age, and comorbidities. The cumulative incidence curves of HZ in both the cohorts were estimated using the Kaplan–Meier analysis, and the difference between the cohorts was compared using the log-rank test. Univariate and multivariate Cox proportional hazards regression models were used to assess the risk of HZ and HZ-associated risk factor. The multivariate model was adjusted for sex, age, comorbidities, and use of steroid. In addition, we compared the hazard ratio (HR) of HZ between both cohorts according to sex, age, and comorbidities. Furthermore, we evaluated the association between the severity of psoriasis and risk of HZ.

A *P* value of less than 0.05 was considered to be statistically significant, and the Statistical Analysis System software (Version 9.4; SAS Institute, Inc., Cary, NC) was used to perform all statistical analyses.

## Results

The psoriasis and nonpsoriasis cohorts consisted of 4077 and 16308 patients, respectively (Table 1). The distribution of sex and age was the same in both cohorts. The average age of the patients in the psoriasis and nonpsoriasis cohorts was 44.64 (SD, 17.34 years) and 44.43 years (SD, 17.45 years), respectively. The prevalence of DM (9.37% vs. 7.06%), hyperlipidemia (14.81% vs. 11.27%), hypertension (23.13% vs. 19.64%), DDCT(1.13% vs. 0.39%), renal disease (6.72% vs. 4.49%), cancer (1.79% vs. 1.31%), COPD (8.71% vs. 6.87), asthma (5.54% vs. 4.12%), and use of steroid (11.5% vs. 5.75%) was higher in the psoriasis cohort than in the nonpsoriasis cohort.

**Table 1:** Baseline demographic factors and comorbidities of study participants.

	Non-psoriasis cohort N = 16308		Psoriasis cohort N = 4077		p-value
	Non-psoriasis cohort N = 16308		Psoriasis cohort N = 4077		p-valu
Variable	n	%	n	%	
Sex					0.99
Women	7232	44.35	1808	44.35	
Men	9076	55.65	2267	55.65	
Age, years					0.99
20–39	7528	46.16	1882	46.16	
40–59	5364	32.89	1341	32.89	
≥ 60	3416	20.95	854	20.95	
Mean (SD)	44.43	(17.45)	44.64	(17.34)	0.50
Comorbidity					
DM	1152	7.06	382	9.37	<0.001
Hyperlipidemia	1838	11.27	604	14.81	<0.001
Hypertension	3203	19.64	943	23.13	<0.001
HF	271	1.66	86	2.11	0.06
Vasculitis	30	0.18	11	0.27	0.37
DDCT	63	0.39	46	1.13	<0.001
Renal disease	733	4.49	274	6.72	<0.001
Cancers	213	1.31	73	1.79	0.02
IBD	109	0.67	38	0.93	0.09
COPD	1121	6.87	355	8.71	<0.001
Asthma	672	4.12	226	5.54	<0.001
Medication					
Steroid use					<0.001
No	15371	94.3	3607	88.5	
Yes	937	5.75	470	11.5	

Abbreviation: SD, standard deviation; DM, diabetes mellitus; HF, heart failure; DDCT, Diffuse diseases of connective tissue; IBD, inflammatory bowel disease; COPD, chronic obstructive pulmonary disease.

**Fig 1:**
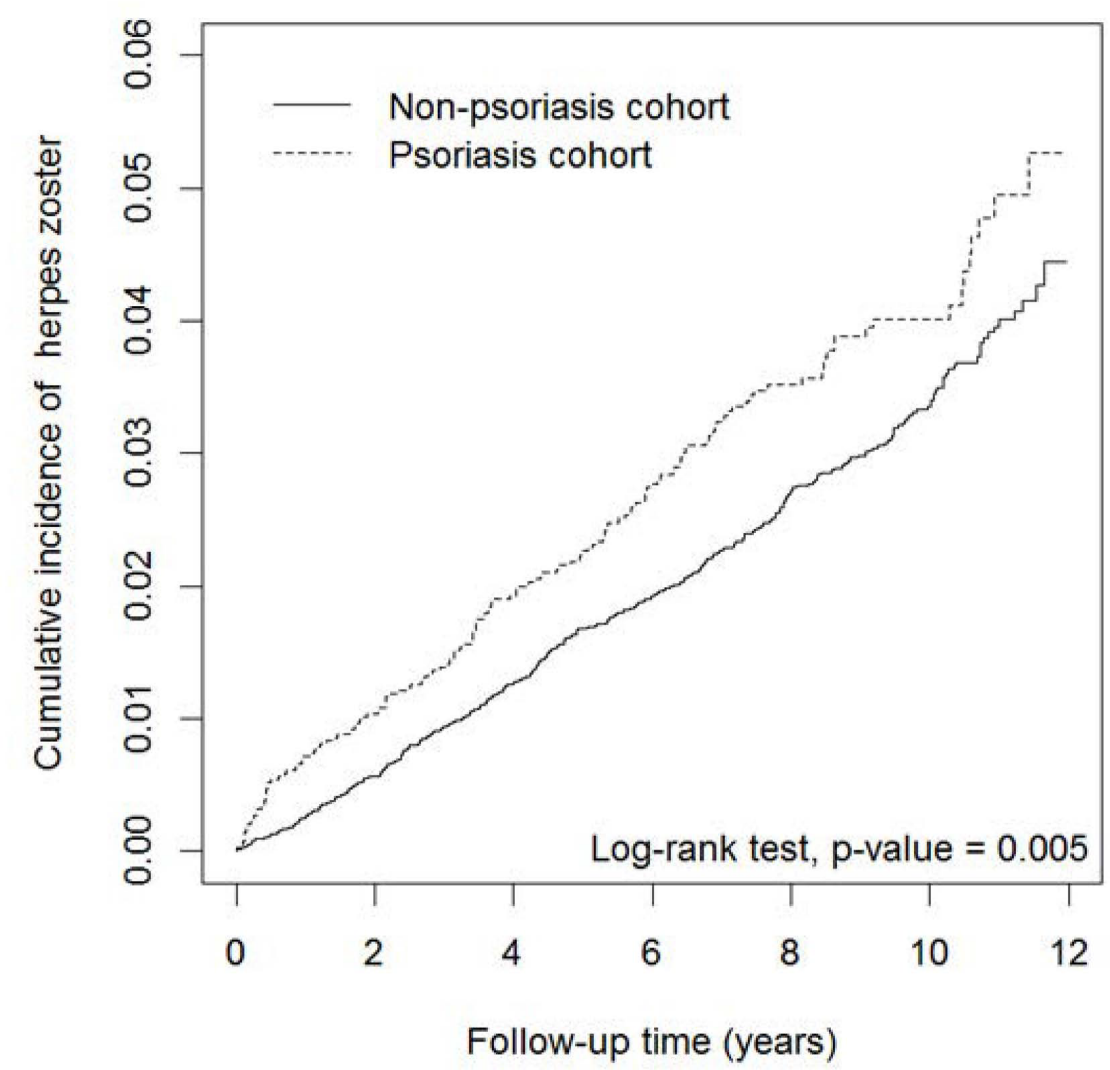
Cumulative incidence curves of herpes zoster in psoriasis and nonpsoriasis cohorts.

The cumulative incidence curves of HZ in the psoriasis and nonpsoriasis cohorts are presented in Fig 1. The cumulative incidence of HZ was significantly higher in the psoriasis cohort than in the nonpsoriasis cohort (log-rank test, p = 0.005).

The mean follow-up duration of the psoriasis and nonpsoriasis cohorts was 7.96 and 7.91 years, respectively. In the psoriasis cohort, 146 patients were diagnosed with HZ, with an incidence density rate of 4.50 per 1000 person-years. In the nonpsoriasis cohort, 444 patients were diagnosed with HZ, with an incidence density rate of 3.44 per 1000 person-years. After adjusting for sex, age, comorbidities, and use of steroid, the risk of HZ was significantly higher in the psoriasis cohort than in the nonpsoriasis cohort (adjusted HR, 1.29; 95% confidence interval [CI], 1.07–1.56; Table 2). Furthermore, the risk of HZ was 2.61-fold (95% CI, 2.05–3.31) and 5.35-fold (95% CI, 4.10–6.97) higher in the patients aged 40–59 years and those aged 60 years and older, respectively, than in the younger patients, indicating that the risk of HZ increases with age. The multivariate analysis revealed that men and use of steroid had a decreased risk of HZ (adjusted HR, 0.81; 95% CI, 0.69–0.96 and adjusted HR, 0.60; 95% CI, 0.44–0.81, respectively); however, the patients with hyperlipidemia had a significantly increased risk of HZ (adjusted HR, 1.36; 95% CI, 1.01–1.68).

**Table 2:** Cox model measured hazard ratios and 95% confidence interval of herpes zoster in association with psoriasis and covariates.

Variable	Event no.	Person-years	IR	HR (95% CI)	
				Univariate	Multivariate^†^
Psoriasis					
No	444	129043	3.44	1.00	1.00
Yes	146	32466	4.50	1.31 (1.08–1.58)**	1.29 (1.07–1.56)**
Sex					
Women	273	73004	3.74	1.00	1.00
Men	317	88505	3.58	0.96 (0.82–1.13)	0.81 (0.69–0.96)*
Age, years					
20–39	107	77741	1.38	1.00	1.00
40–59	210	54338	3.86	2.81 (2.23–3.55)***	2.61 (2.05–3.31)***
≥ 60	273	29430	9.28	6.82 (5.45–8.53)***	5.35 (4.10–6.97)***
Comorbidity					
DM					
No	499	151097	3.30	1.00	1.00
Yes	91	10412	8.74	2.68 (2.14–3.35)***	1.18 (0.92–1.51)
Hyperlipidemia					
No	440	143511	3.07	1.00	1.00
Yes	150	17998	8.33	2.74 (2.27–3.29)***	1.36 (1.01–1.68)**
Hypertension					
No	352	132075	2.67	1.00	1.00
Yes	238	29434	8.09	3.06 (2.59–3.60)***	1.19 (0.96–1.46)
HF					
No	564	159520	3.54	1.00	1.00
Yes	26	1989	13.07	3.78 (2.55–2.60)***	1.35 (0.89–2.04)
Vasculitis					
No	585	161220	3.63	1.00	1.00
Yes	5	288	17.34	4.85 (2.01–11.69)***	2.07 (0.85–5.04)
DDCT					
No	583	160783	3.63	1.00	1.00
Yes	7	726	9.65	2.70 (1.28–5.69)**	2.01 (0.95–4.27)
Renal disease					
No	537	154808	3.47	1.00	1.00
Yes	53	6700	7.91	2.30 (1.74–3.05)***	1.05 (0.78–1.42)
Cancers					
No	579	159800	3.62	1.00	1.00
Yes	11	1709	6.44	1.80 (0.99–3.27)	0.97 (0.53–1.76)
IBD					
No	582	160491	3.63	1.00	1.00
Yes	8	1018	7.86	2.22 (1.10–4.45)*	1.34 (0.66–2.69)
COPD					
No	501	151722	3.30	1.00	1.00
Yes	89	9786	9.09	2.79 (2.22–3.49)***	1.24 (0.96–1.62)
Asthma					
No	539	155484	3.47	1.00	1.00
Yes	51	6025	8.46	2.48 (1.86–3.30)***	1.27 (0.93–1.75)
Medication					
Steroid use					
No	541	150333	3.60	1.00	1.00
Yes	49	11176	4.38	1.22 (0.91–1.63)	0.60 (0.44–0.81)***

Abbreviation: IR, incidence density rate per 1000 person-years; HR, hazard ratio; CI, confidence interval; DM, diabetes mellitus; HF, heart failure; Diffuse diseases of connective tissue DDCT, mixed connective tissue disease; IBD, inflammatory bowel disease; COPD, chronic obstructive pulmonary disease.

^†^ Adjusted for psoriasis, sex, age (categorical), diabetes mellitus, hyperlipidemia, hypertension, heart failure, vasculitis, mixed connective tissue disease, renal disease, cancer, IBD, COPD, asthma, and steroid use in Cox proportional hazards regression.

* *P* < .05

** *P* < .01

*** *P* < .001

Sex stratification revealed that compared with the women without psoriasis, the women with psoriasis had an increased risk of HZ (adjusted HR, 1.36; 95% CI, 1.04–1.79). However, compared with the men without psoriasis, the men with psoriasis had a null risk of HZ (adjusted HR, 1.21; 95% CI, 0.94–1.58; Table 3). Furthermore, age stratification revealed that the risk of HZ was higher in the patients with psoriasis than in the patients without psoriasis in the age group of 20–39 years (adjusted HR, 1.77; 95% CI, 1.17–2.66). In individual without any comorbidities, patients with psoriasis had a higher risk of HZ than in subjects without psoriasis (adjusted HR, 1.37; 95% CI, 1.02–1.84).

**Table 3:** Incidence density rates and hazard ratios of herpes zoster according to the psoriasis status stratified by sex, age, and comorbidities.

	Psoriasis						Compared to non-psoriasis cohort	
	No			Yes			HR (95% CI)	
Variable	Event no.	Person-years	IR	Event no.	Person-years	IR	Crude	Adjusted^‡^
Sex								
Women	202	58312	3.46	71	14692	4.83	1.39 (1.06–1.83)*	1.36 (1.04–1.79)*
Men	242	70732	3.42	75	17773	4.22	1.23 (0.95–1.60)	1.21 (0.94–1.58)
Age, years								
20–39	73	61969	1.18	34	15772	2.16	1.83 (1.22–2.74)**	1.77 (1.17–2.66)**
40–59	165	43458	3.80	45	10880	4.14	1.09 (0.78–1.51)	1.03 (0.74–1.44)
≥ 60	206	23617	8.72	67	5813	11.53	1.32 (1.01–1.74)*	1.32 (0.99–1.74)
Comorbidity status^†^								
No	190	92685	2.05	57	21621	2.64	1.28 (0.95–1.72)	1.37 (1.02–1.84)*
Yes	254	36358	6.99	89	10845	8.21	1.18 (0.92–1.50)	1.26 (0.99–1.60)

Abbreviation: IR, incidence density rate per 1000 person-years; HR, hazard ratio; CI, confidence interval.

^†^ Patients with diabetes mellitus, hyperlipidemia, hypertension, heart failure, vasculitis, mixed connective tissue disease, renal disease, cancer, IBD, COPD, or asthma were classified as the comorbidity group.

^‡^ Mutually adjusted for sex, age (continuous), diabetes mellitus, hyperlipidemia, hypertension, heart failure, vasculitis, mixed connective tissue disease, renal disease, cancer, IBD, COPD, asthma, and steroid use.

* *P* < .05

** *P* < .01.

Furthermore, we divided the psoriasis cohort into 2 subgroups according to their prescribed treatment. We observed that the risk of HZ was significantly higher in the patients with psoriasis who received immunomodulatory therapy or phototherapy (severe psoriasis) than in those without psoriasis (adjusted HR, 1.61; 95% CI, 1.15–2.27; Table 4).

**Table 4:** Incidence density rates and hazard ratios of herpes zoster in different psoriasis severitie.

Subgroup	N	Event no.	Person-years	IR	HR (95% CI)	
					Crude	Adjusted^‡^
Non-psoriasis cohort	16308	444	129043	3.44	1.00	1.00
Psoriasis cohort						
Mild psoriasis	3233	109	25575	4.26	1.24 (1.01–1.53)*	1.20 (0.97–1.48)
Severe psoriasis^†^	844	37	6891	5.37	1.55 (1.11–2.17)*	1.61 (1.15–2.27)**

Abbreviation: IR, incidence density rate per 1,000 person-years; HR, hazard ratio; CI, confidence interval.

^†^ Severe psoriasis was defined by received treatment involving phototherapy (including ultraviolet B, or psoralen and ultraviolet A) and/or immunomodulator drugs (including methotrexate, azathioprine, ciclosporin, oral retinoids, hydroxyurea, mycophenolate mofetil, tacrolimus, etanercept, adalimumab and ustekinumab).

^‡^ Adjusted for sex, age (continuous), diabetes mellitus, hyperlipidemia, hypertension, heart failure, vasculitis, mixed connective tissue disease, renal disease, cancer, IBD, COPD, asthma, and steroid use.

* *P* < .05

** *P* < .01

## Discussion

In our study population, the risk of HZ was higher in women, elderly patients, patients with psoriasis, and patients with comorbid hyperlipidemia. Furthermore, a comparison between the psoriasis and nonpsoriasis cohorts revealed that the risk of HZ was higher in women and younger patients (aged between 20 and 39 years). Although the risk of HZ was higher in elderly patients than in younger patients, the difference between the cohorts was not significant. The results demonstrated that the risk of HZ was higher in the severe psoriasis cohort than in the nonpsoriasis cohort. Moreover, compared with the nonpsoriasis cohort, HZ was significantly associated with psoriasis in the severe psoriasis cohort. The severity of psoriasis may indicate the intrinsic factors that affect the incidence of HZ. We classified patients who received aggressive therapy, including systemic or biology therapy or phototherapy as having severe psoriasis and who were not prescribed systemic or biology therapy or phototherapy for psoriasis were classified as having mild psoriasis. Therefore, systemic therapy may have a major role in increasing the risk of HZ in our study. However, we cannot exclude the influence of the intrinsic factors of psoriasis.

Studies investigating drug-related HZ in patients with psoriasis have discussed various antipsoriatic medications such as methotrexate, cyclosporine, phototherapy, oral retinoids, and biological agents. Dreiher et al. reported that HZ was significantly associated with infliximab but not with biological agents.[9] Moreover, a study conducted in 2015 that used a database from Israel reported no risk of HZ when a single agent was used; however, the risk was significantly higher when a combination of biological agents and methotrexate was used. By contrast, acitretin reduced the incidence of HZ.[10] Furthermore, infliximab, one of the most commonly used tumor necrosis factor-alpha (TNF-α) inhibitors, increased the risk of HZ in Adelzadeh et al.’s study, whereas it reduced the risk of VZV reactivation in El Hayderi et al.’s study.[14, 15] However, the HZ incidence in patients with psoriasis receiving treatment with adalimumab, etanercept, and ustekinumab remained unclear.[14] Two studies have investigated the association of efalizumab with HZ in results of less than 2% and have reported a crude HZ incidence rate of 28 per 1000 patients.[16, 17] In addition to patients with psoriasis, some studies have evaluated the association of HZ with biological agents in all patients receiving biological agents. Winthrop et al. reported that among patients with inflammatory diseases, the risk of HZ was higher in patients who received anti-TNF therapies than in those who received nonbiological treatments.[18] Furthermore, Failla et al. reported that the HZ incidence was 2.1-fold higher in patients aged more than 60 years receiving biological agents; however, it was not significant in patients receiving ustekinumab.[19, 20] According to a systemic review conducted in patients with rheumatoid arthritis, evidence on the association of methotrexate with varicella or HZ has been lacking.[21] Therefore, the risk of HZ because of the usage of antipsoriatic agents remains controversial. Moreover, no association of biological agents and methotrexate with HZ has been reported. The increased risk of HZ in patients receiving a combination of biological agents and methotrexate can be because of the additive immunosuppressive effect or drug interaction leading to increased medication levels in plasma.[10]

TNF-α is a pivotal proinflammatory cytokine that amplifies inflammation. The downregulation of interferon gamma (IFN-γ) after the blocking of TNF-α signaling pathways is hypothesized to exacerbate VZV dissemination.^[^^22^^]^ Winthrop et al. reported that the crude incidence rate of HZ among anti-TNF users is 4.4 per 1000 patient-years for autoimmune diseases such as psoriasis, psoriatic arthritis, and ankylosing spondylitis.[18] However, studies on autoimmune diseases have confirmed the counteraction in the cross-regulation between TNF-α and IFN-α; that is, TNF-α antagonist use results in the overexpression of IFN-α-regulated genes in plasmacytoid dendritic cells (pDC), which presumably facilitates the antiviral reaction.[23, 24] IFN-α secreted by pDC participates in both the antiviral reaction and immune response and may have a major role in the association of viral infections with TNF-α-induced autoimmune diseases. This may explain the different outcomes of previous studies on TNF-α antagonists and HZ flare-up. Compared with patients without psoriasis, the risk of VZV flare-up was significantly increased in patients with mild psoriasis who did not receive systemic therapy. Thus, the pathophysiology of psoriasis may be associated with that of subsequent HZ.

The tropisms of VZV in the T cells and skin have been extensively studied. First, VZV triggers the activation of naive T cells and promotes the replication and release of infectious virions in all CD3+ T cells, including CD4+ and CD8+ T cells.[25] Then, VZV-infected CD4+ T cells mainly exhibit a memory T-cell phenotype and express activation markers and skin-homing proteins such as cutaneous leukocyte antigen (CLA) and C-C chemokine receptor 4.^[^^26^^]^ Moreover, the recruitment of CD4+ T cells to the skin in patients with psoriasis has been extensively investigated. Similarly, the homing of CD8+ T cells into the human skin mediated by CXCL16–CXCR6 interactions is equally crucial.[27–30] We suppose that the potential HZ outbreak in the psoriatic skin is related to the recruitment of CLA+ T cells. Further investigation is required to clarify the actual mechanism. Another similar characteristic between patients with psoriasis and those with HZ is plasma vitamin D levels that are lower than the normal range.[31] Chao et al. reported a positive association of serum vitamin D levels with immunity against VZV and illustrated immunomodulatory effects provided by the binding of vitamin D receptors to neutrophils, T cells, and antigen-presenting cells.[32, 33] Although vitamin D is used as a guideline therapy in patients with psoriasis, its immune reactions that contribute to the development of subsequent HZ should be considered.

From the psychosocial viewpoint, depression and anxiety more frequently occur in women and anxiety more often occurs in younger patients with dermatological diseases. Studies have reported that among all dermatological diseases, the percentage of depression and anxiety is the highest in psoriasis.[34, 35] The younger patients usually focus more attention on their appearance; hence, they may more frequently seek help for their cosmetic problems. These results are consistent with our findings that the HZ incidence was higher in women and younger patients. In addition, psychological stress is a risk factor for HZ infection.[36] Furthermore, a cross-sectional multicentre study conducted in 13 European countries suggested that among all dermatological diseases, the suicidal ideation rate was the highest in psoriasis.[37] This indicates that psychological stress in patients with psoriasis may lead to a vicious cycle and further deteriorate the disease.[38]

The strength of our present study is that its design facilitated the comparison of the risk of VZV dissemination between patients with psoriasis receiving and not receiving systemic therapy. Moreover, the NHIRD provided the data with comprehensive medical history, good reliability, and nationwide coverage. However, our study has some limitations. First, not all antipsoriatic medications are available in Taiwan. For example, efalizumab and infliximab, which have been commonly discussed in previous studies, are not included in the NHIRD. A lack of these data might deviate the statistical results. Second, because of the high cost of biological agents, few patients received systemic therapy before 2009. Since 2009, biological agents have been covered by the health insurance with conditions including failure after the thorough treatment with first-line systemic agents. Therefore, in our follow-up period (2000–2011), some differences were observed in medication usage. A follow-up study with different medications should be conducted in the future. Third, we did not include topical corticosteroids because of the presence of bias related to over-the-counter medication and difficulty in control dosage. Even we cannot exclude the influence of topical corticosteroids on epidermal cells (eg. Langerhans cells and keratinocytes) regarding immunosuppressive effects leading to VZV reactivation. Our result indicates corticosteroids usage does not increase the risk of HZ. Moreover, although the patients with HZ definition was subjects without concurrent received antiviral medication, several related publications about HZ using the same operational definition from our research team. [39, 40] Nevertheless, in this study, the diagnoses of HZ were based on the ICD-9-CM codes, as determined by qualified clinical physicians) for the strictly audited reimbursement process. Furthermore, NHIRD covers a highly representative sample of Taiwan’s general population because the reimbursement policy is universal and operated by a single-buyer, the Taiwanese government. All insurance claims are scrutinized and coded by medical reimbursement specialists and peer reviewed according to the standard criteria for diagnoses in the study. Moreover, incorrect diagnoses or coding mistakes result in considerable penalties for the physicians. The reliability and validity of the NHI research database for epidemiologic investigations have been reported previously. Therefore, the diagnoses and coding in this study should be highly reliable.

Our results demonstrated a higher risk of HZ in the psoriasis population. Identifying individuals who are particularly vulnerable to opportunistic infections can help physicians. Insights of HZ infection in immunocompromised patients could help to prevent the incidence by using a vaccine. Our findings can help policymakers in proposing specialized protection policies for patients with psoriasis. Additional studies evaluating the effect of antipsoriatic medications and the molecular mechanisms of the intrinsic factors of psoriasis underlying the association of psoriasis with HZ should be conducted.
